# Novel computer-based assessments of everyday visual function in people with age-related macular degeneration

**DOI:** 10.1371/journal.pone.0243578

**Published:** 2020-12-07

**Authors:** Bethany E. Higgins, Deanna J. Taylor, Wei Bi, Alison M. Binns, David P. Crabb

**Affiliations:** Optometry and Visual Sciences, School of Health Sciences, University of London, London, United Kingdom; University of Florida, UNITED STATES

## Abstract

**Purpose:**

To test the hypothesis that the performance in novel computer-based tasks of everyday visual function worsens with disease severity in people with non-neovascular age-related macular degeneration.

**Methods:**

Participants with and without non-neovascular age-related macular degeneration (≥60 years, minimum logMAR binocular visual acuity 0.7) performed a series of standard visual function tests and two novel computer-based tasks. In a visual search task, participants had to locate an image of a single real-world object within an array of 49 distractor images. Next, in a series of simulated dynamic driving scenes, participants were asked to identify one or two approaching real-world road signs and then select these road signs from four options. Outcome measures were median response times and total correct responses.

**Results:**

Forty-nine participants had no macular disease (n = 11), early/intermediate age-related macular degeneration (n = 16) or geographic atrophy (n = 22). Groups were age-similar with median (interquartile range) logMAR visual acuity of 0.00 (-0.08,0.12), 0.13 (-0.08,0.70) and 0.32 (0.12,0.70) respectively. Median (interquartile range) visual search response times were 1.9 (1.0,2.4), 1.8 (1.1,3.7) and 2.4 (1.2,6.0) seconds respectively. Median (interquartile range) road sign response times (single road signs) were 1.2 (0.4,1.7), 1.5 (0.9,2.8) and 1.8 (1.0,5.5) seconds respectively. Median (interquartile range) road sign response times (double road signs) were 1.7 (0.7,2.4), 2.3 (1.2,3.1) and 2.5 (1.7,6) seconds respectively. Participants with geographic atrophy recorded slower response times in all tasks and over 50% performed outside the normative limit for task performance. There were no significant differences between groups in total correct responses across all tasks.

**Conclusions:**

In a novel computer-based assessment, people with increasing severity of age-related macular degeneration take longer to perform visual search of everyday objects and take longer to identify road signs than those with no age-related macular degeneration. These novel assessments could be useful as patient-relevant, secondary outcomes for clinical trials.

## Introduction

Age-related macular degeneration (AMD) is the most common cause of visual impairment in the ageing population of developed countries [1]. Dry AMD (non-neovascular), which is responsible for the vast majority of AMD cases, can typically lead to slow, irreversible loss of vision [2]. Subsequent visual impairment associated with AMD compromises quality of life and reduces independence [3]. Due to central vision loss, everyday visual activities can be made difficult and can eventually become impossible [4]. Standard clinical tests, like measures of visual acuity (VA), may not capture the nuances of these visual functions, such as for example finding something on a supermarket shelf or detecting a road sign. Developing methods to easily measure everyday visual function is the subject of the work reported in this paper, with the long-term aim of developing assessments that could be used as meaningful secondary outcome measures for clinical trials for future potential treatments for AMD.

Looking for something (visual search) is a ubiquitous everyday activity. Visual search is important to people and we have previously shown this can be measured on a computer-based task [5, 6]. For example, we have previously demonstrated that people with AMD, certainly those with advanced disease, have measurable difficulties beyond those observed in visually healthy peers on a computer-based visual search task [5]. Instead of asking participants to find synthetic targets or optotypes, as in the case with many studies [7, 8], we used images of everyday scenes. The small number of studies that have recruited people with AMD and conducted real-world visual search tasks were primarily focussed on eye movements [9–11] and not outcomes such as response time.

Visual search is of course, utilised in driving. We reported that a group of people with AMD that performed significantly slower in a visual search task compared to visually healthy controls had a median VA within legal driving standards [5]. Hence, this finding may have implications on driving safety in people with AMD as it may take individuals longer to react to seeing an impending hazard or important road sign, despite being deemed safe to drive by the United Kingdom (UK) government guidelines [12, 13].

Investigating the association between vision loss from AMD and driving performance is not straightforward. For example, older drivers with intermediate AMD have been shown to have reduced risk of collision involvement but this might be due to self-regulatory behaviour [14]. Recently, Wood et al. (2018) assessed people with AMD and peers with normal vision under in-traffic conditions in an automatic, dual-brake vehicle. Interestingly, those with AMD struggled with effectively scanning road scenes and subsequently made more observation-based errors [15]. An overarching link between AMD and poorer driving performance is unsubstantiated in the literature [15]. Moreover, as ageing populations continue to rise, discussions on driving, VA and people with AMD will become more clinically and legally essential [16]. We feel that real-world assessments of vision used in driving are pertinent in understanding the impact of AMD on everyday visual tasks.

In this study we investigated computer-based real-world visual search performance in people with AMD, using two novel assessments. First, using a collage of images of everyday objects to mimic visual search in a busy environment. Second, a road sign detection task to imitate the comprehension of approaching road signs while in a moving vehicle. We hypothesise that the performance on these tasks varies between people classified to be at different stages of AMD severity.

## Methods

### Study participants

We conducted a cross-sectional study as part of a programme of work described elsewhere [5, 17]. Individuals with dry AMD were recruited from Moorfields Eye Hospital Trust, London, optometrists local to City, University of London and the membership of the Macular Society (www.macularsociety.org). Our eligibility criteria required participants to be aged ≥60 years, have adequately clear ocular media, sufficient pupillary dilation and fixation to allow quality fundus imaging (Lens Opacities Classification System [LOCS] III grading scale [18] of grade <3), and to have dry AMD (early/intermediate/late) in their better-seeing eye (assessed by best-corrected VA [BVA]). Fellow eyes of individuals with dry AMD were allowed to have any AMD status. BVA was required to be 0.7 logMAR or better (Snellen equivalent of 6/30) as measured using an Early Treatment Diabetic Retinopathy Study (ETDRS) chart. AMD participants were excluded if they had wet AMD in their better-seeing eye, had any other ocular or systemic diseases that could affect visual function or history of any medication known to alter macular function (e.g. tamoxifen or chloroquine), or high risk of angle closure during pupillary dilation (Van Herick <Grade 2, history of angle closure or experience of prodromal symptoms of angle closure). In addition, participants were required to pass an abridged version of the Mini Mental State Evaluation (MMSE) [19] to screen for cognitive function. Lastly, participants had to have a sufficient understanding of the English language to understand the Participant Information Sheet, history and symptoms questioning and test instructions.

Visually healthy controls were recruited from the City Sight Optometry Clinic at City, University of London. People attending this clinic for eye examinations are invited to sign-up to be contacted if they wish to be recruited for research studies for which they might be a potentially suitable participant. Eligibility criteria for controls was the same as for people with AMD except participants were required to have no AMD (or any other eye disease) in either eye, and monocular VA of 0.3 logMAR (6/12) or better.

The study was approved by Nottingham 2 National Health Service (NHS) Research Ethics Committee and was conducted according to the tenets of the Declaration of Helsinki (IRAS ID: 166958). Written, informed consent was obtained from each participant prior to examination. Participant information was anonymised before being entered into a secure computer database.

### Clinical examination and screening

After providing informed consent, participants underwent a series of baseline examinations to evaluate their AMD status and to ensure eligibility for participation, conducted by an optometrist (DJT). Medical history and symptoms were taken including questions from the EQ-5D questionnaire. The EQ-5D questionnaire is a patient reported outcome measure (PROM) used to determine general health status using five domains: mobility, self-care, usual activities, pain/discomfort, and anxiety/depression [20]. This PROM gives an overall index score of general health calculated by deducting the appropriate weights of each domain from 1, the value for full health. Best corrected VA was determined via a backlit ETDRS chart (mean luminance of 203.5 cd m-2) at 4 metres (mono- and binocularly). Contrast sensitivity (CS) was tested with the Pelli-Robson chart at 1 metre (binocularly) with best-corrected distance prescription, scored per-letter [21].

Following the study tests, participants underwent dilated fundus examination. Lens clarity was graded using the slit lamp biomicroscope, according to the LOCS III grading scale [18]. Microperimetry (MP) was performed using the MAIA microperimeter (CenterVue, Padova, Italy). A total of 37 points in a radial test pattern were tested over the central 10° of the retina, measured using white Goldmann III targets presented for 200ms, and thresholds were calculated using the system’s full threshold 4–2 staircase strategy. In addition, fundus images were obtained including digital colour fundus photography, spectral-domain optical coherence tomography, and fundus autofluorescence. These were collectively used to classify and grade AMD status by the better-seeing eye (determined by VA) as early, intermediate, or late according to the Beckman classification scale [22]. This widely used scale grades macular disease status according to drusen size, pigmentary abnormalities, and presence or absence of geographic atrophy (GA) or neovascular AMD.

### Study procedure

Participants were seated 50 cm away from a touch screen Dell 23 inch touch monitor—P2314T (resolution 1920×1080), subtending an approximate visual angle of 54° horizontally and 32° vertically. Participant head position was not fixed in order for the test to be as natural as possible, although viewing distance was visually monitored and participants were reminded to not to move closer or further away from the touch screen monitor. Tests were performed binocularly using habitual refractive correction. Participants were able to move their eyes freely and responded to the below tasks by touching the screen and the space bar on the keyboard. Tests were conducted in a darkened room to increase contrast of the computer screens from the surrounding environment.

To account for response time (RT) differences between participants, a baseline RT was calculated during the preliminary section of the tasks. The average time taken to touch the monitor in absence of a detailed visual stimulus was recorded over 40 trials. This median baseline RT was subtracted from the median task RTs of each participant to give a calibrated RT (S1 Video).

### Visual search task procedure

**Fig 1:**
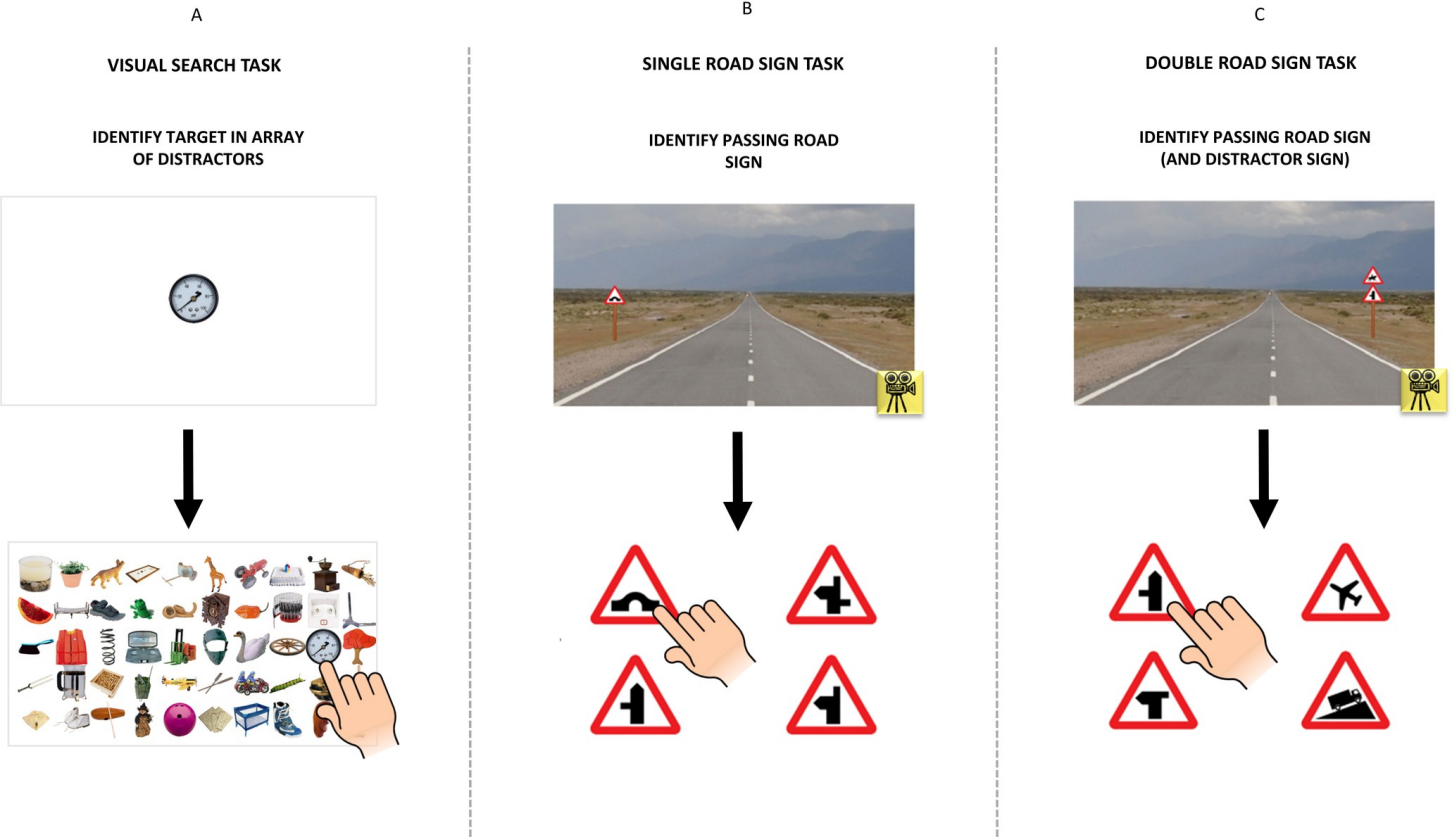
Task procedures. A schematic showing the task procedures for the visual search task (A) and both road sign tasks (B-C).

For each trial, the participants were shown a random image of an everyday object in the centre of the touch screen monitor for 0.5 seconds. These images were sourced from a previously described database of 2400 everyday objects [23]. Participants were instructed by text on the screen to touch the space bar when they were ready to select the image they had been shown. The participants were then required to find and touch this target image as quickly as possible. The participants did not receive feedback if they were correct or not. The target image was within a 10x5 array of 49 different images of everyday objects, spaced uniformly. The target image was randomly arranged at different locations within the array for each trial. There were eight practice trials, followed by 40 test trials. (See Fig 1 and S2 Video). Note, the target image shown at the start was the same size as the target image embedded in the array.

### Road sign identification task procedures

There were two parts to the road sign identification task: single road signs and double road signs (see Fig 1). During a trial of the single road sign task, participants were shown a dynamic scene of a road in which they ‘approached’ a randomly selected road sign. All road signs included were from the UK Highway Code [13] and could appear on either side of the road. Participants were instructed to press the space bar when they identified the road sign approaching. Immediately, the screen changed from the road scene to a presentation of four different road signs, including the correct target sign identified previously. Participants made their choice by touching the sign on the screen. There were 12 practice trials to begin with and then 40 test trials. (See S3 Video).

The double road sign task simply differed by presenting two road signs: the target image, and a distractor. The target could be the upper or lower sign and the sign could appear on either side of the road. As before, participants were instructed to press the space bar when they identified the road signs and then choose (touch screen) the correct sign from four options. The distractor was not one of the options. There were 12 practice trials to start and then, as with the other tasks, 40 test trials. (See S4 Video).

For each of the three tasks (Fig 1), total correct responses (TCR) out of 40 were recorded. RTs, calibrated by the baseline measure and censored at six seconds, were also recorded for each trial. Median RT over all 40 trials was the main outcome measure for each person for each the three tests. The median was chosen as a robust measure of overall person response.

### Data analysis

A 90% normative reference limit was calculated from the sample of the median RTs from the control group. Due to the data being skewed, this limit was estimated by a direct percentile method [24] using the formula *n = (90/100) x N*, where N = number of ordered (smallest to largest) values in the sample and n = ordinal rank of a given value. Median RTs for the AMD groups were then compared to this set ‘normative’ limit. The same analysis was conducted for the TCRs.

Average differences in test parameters between AMD severity groups were analysed with a Kruskal-Wallis test and a post hoc analysis (Dunn’s test), adjusted for by Bonferroni correction. Simple univariate association (Spearman's rank correlation coefficient) were evaluated between outcome measures and visual function measures (VA, CS and MP measures [for the better and worse eye]), and other factors such as age and EQ-5D index scores. (Non-parametric tests were chosen as the distribution of data were skewed.)

Statistical analysis was done in R 3.5.2 (http://www.r-project.org/) under RStudio, version 1.1.463 (RStudio, Boston, MA, USA) [25]. Plots were constructed using the ggplot2 packages within R.

## Results

### Demographic and clinical data

Forty-nine participants (82% female) were included and stratified, according to the Beckman classification scale using the better eye [22], into three groups: visually healthy (n = 11; age-related), early/intermediate AMD (n = 16) and GA (n = 22). The groups were age similar and the participants were in general good health (Table 1).

**Table 1:** Median (interquartile range; IQR) demographic and clinical data of participants.

	Control (11)	Early/Intermediate AMD (16)	Geographic Atrophy (22)
**Age (years)**	75 (63, 81)	78 (61, 87)	76 (65, 86)
**Duration of diagnosis (months)**	N/A	45 (24,120)^a^	50 (8, 360) ^a^
**Visual Acuity (logMAR)**	.00 (-.08, .12) ^b^	.13 (-.08, .7)	.32 (.12, .7) ^b^
**Contrast Sensitivity (logCS)**	1.95 (1.6, 1.95)	1.65 (1.05, 1.95)	1.55 (.3, 1.95) ^c^
**EQ-5D (index)**	0.81	0.88	0.82

^a^ Two Early/Intermediate and one GA participant’s AMD duration data was incomplete thus excluded.

^b^ One Control and one GA participant’s visual acuity data was incomplete thus excluded.

^c^ One GA participant’s contrast sensitivity data was incomplete so excluded.

There was no statistically significant variation of baseline response time between the participant groups (Kruskal-Wallis; *P* = .40).

### Visual search

For median RT for each individual’s 40 trials, 17 people with AMD (3 from early/intermediate group and 14 from the GA group [44% of patients]) exceeded the 90% normative limit set by the controls. There was a statistically significant difference in median RT between the groups (Kruskal-Wallis test; *P* = .007). The GA group’s median RTs were significantly slower than the control group (*P* = .030) and the early/intermediate group (*P* = .023) when compared with post-hoc tests. However, the early/intermediate group’s median RTs were not significantly slower than the control group. For TCR, 16 people with AMD (5 from early/intermediate group and 11 from the GA group [42% of patient cohort]) exceeded the 90% normative limit set by the controls. However, there was no evidence of a statistically significant difference between median TCRs between groups (*P* = .342).

### Single road sign

For RT, 19 people with AMD (6 from early/intermediate group and 13 from the GA group [50% of AMD cohort]) exceeded the 90% normative limit. There was a statistically significant difference in median RT between the groups (Kruskal-Wallis test; *P* = .004). The GA group’s median RTs were significantly slower than the control group’s (*P* = .003) but not significantly slower than the early/intermediate group’s RTs (*P* = .400) compared with post-hoc tests. The early/intermediate group was not significantly slower than the control group’s median RTs. For TCRs, 10 people with AMD (1 from early/intermediate group and 9 from the GA group [26% of patient cohort]) exceeded the 90% normative limit. There was a statistically significant difference between the groups (Kruskal-Wallis test; *P* = .030). However, there were no significant differences between TCRs once adjusted for multiple testing.

### Double road sign

For RTs, 23 people with AMD (9 from early/intermediate group and 14 from the GA group [61% of patient cohort]) exceeded the 90% normative limit. There was a statistically significant difference between the groups (Kruskal-Wallis test; *P* = .004). Post hoc analysis indicated GA group’s median RTs were significantly slower than the control group (*P* = .003). Although, there was no evidence of a significant difference between the early/intermediate group and the GA group’s median RTs and the early/intermediate group and the control group’s median RTs. For TCRs, 7 people with AMD (3 from early/intermediate group and 4 from the GA group [18% of patient cohort]) exceeded the 90% normative limits. There was no significant difference between the TCRs of all groups (*P* = .433).

**Fig 2:**
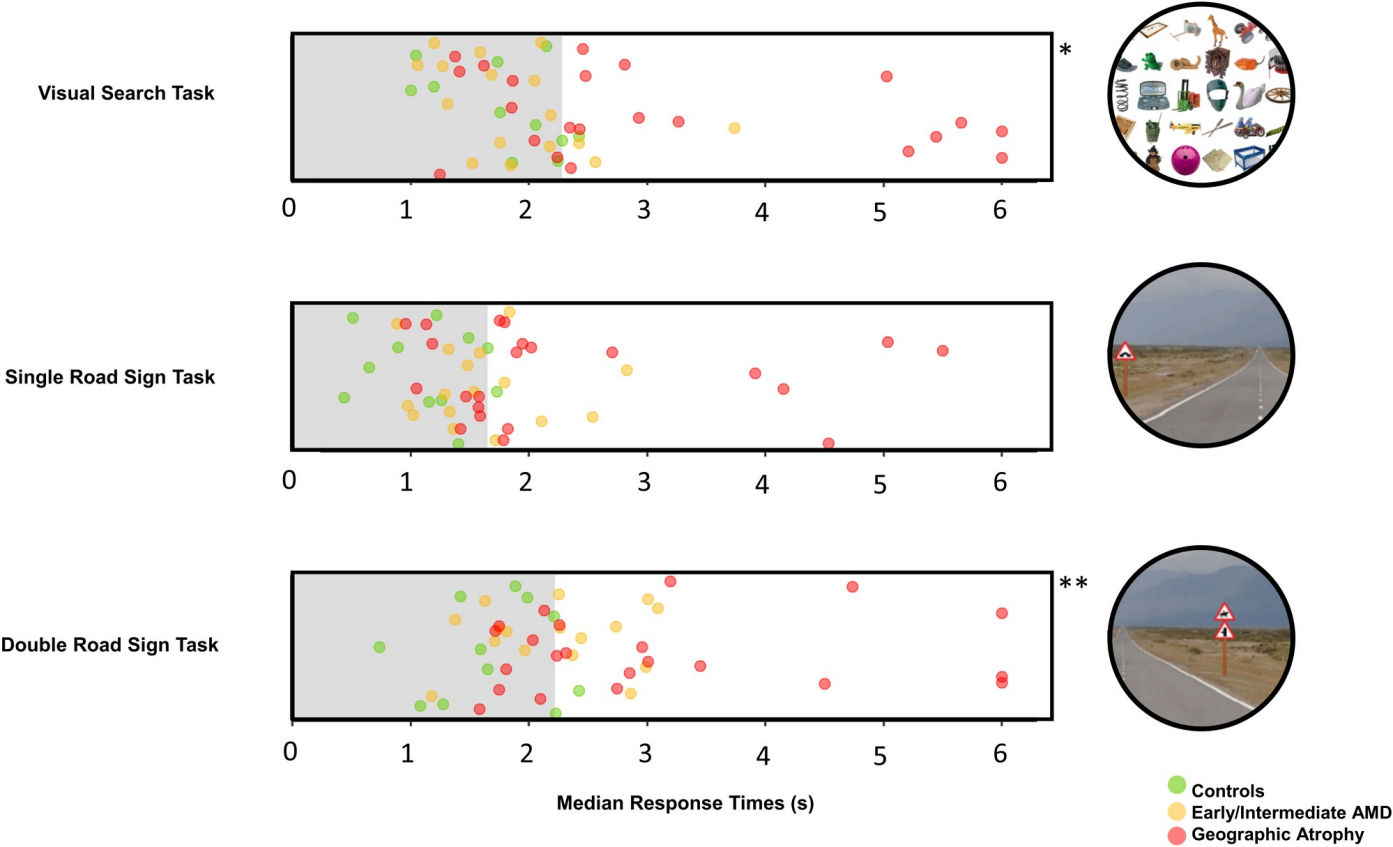
Plot of median response times. Median response times (seconds) capped at 6 seconds for the three participant cohorts with respect to the normative limit, shown as the shaded area. The normative limit for the visual search task was 2.28s, for the single road sign task was 1.65s and for the double road sign task was 2.22s. *The original RTs for two GA group participants whom were capped at 6 seconds were 8.5 and 11.4 seconds. ** The original RTs for three GA group participants whom were capped at 6 seconds were 7.4, 7.0 and 6.6 seconds.

**Fig 3:**
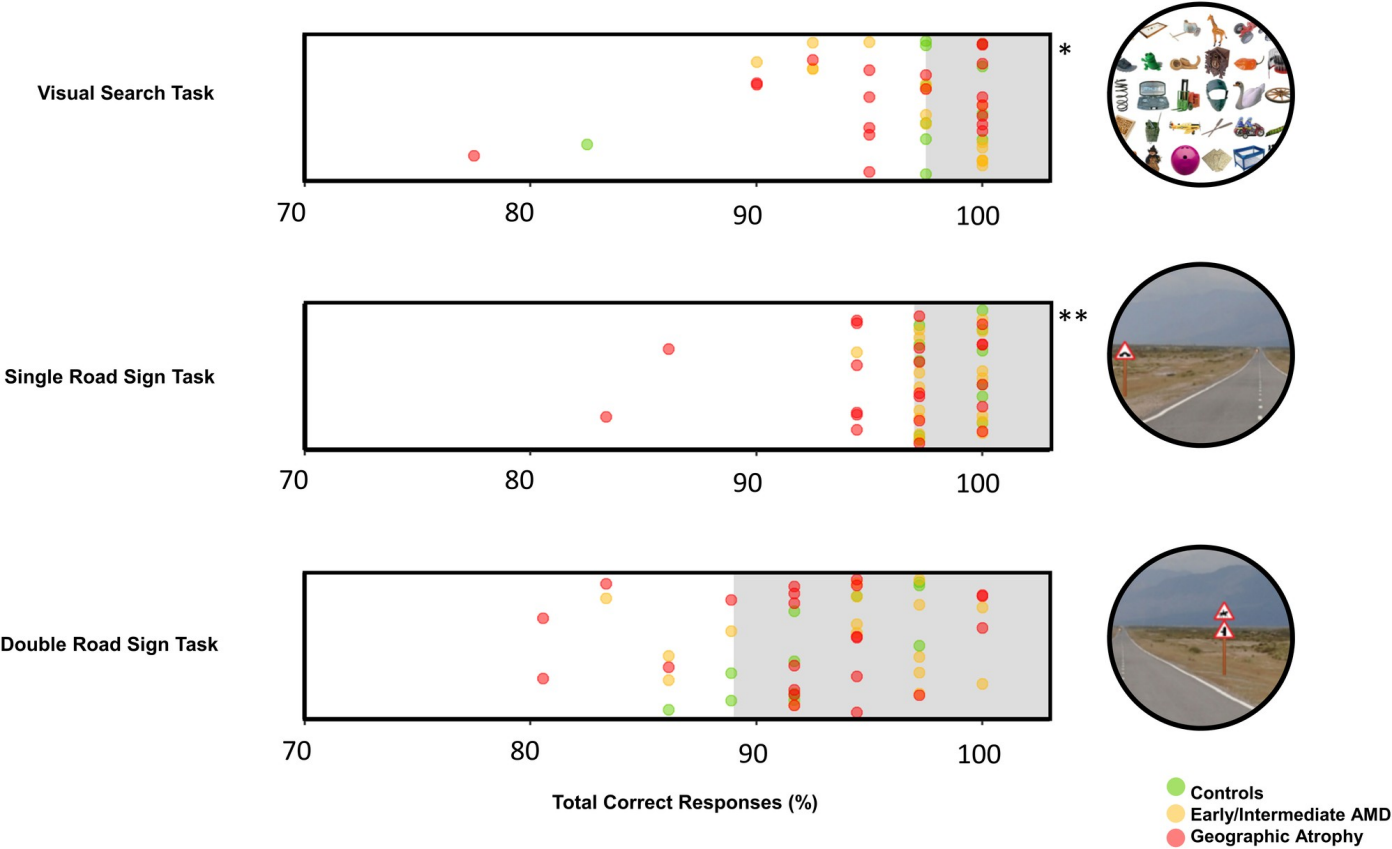
Plot of median total correct responses. The total correct responses (%) of the three participant cohorts with respect to the normative limit shown as the shaded area. The limit for the visual search task was 98%, for the single road sign task was 97% and for the double road sign task was 89%. *Two participants from the GA group have been omitted as the axis was truncated at 70%. TCRs were 13% and 33%. **One participant from the GA group was omitted, TCR was 56%.

Results for all three tasks are further summarised Table 2 and Figs 2 and 3.

**Table 2:** Median (IQR) task response times (seconds; s) and total correct responses.

	Control (11)		Early/Intermediate AMD (16)		Geographic Atrophy (22)	
	RT (s)	TCR (%)	RT (s)	TCR (%)	RT (s)	TCR (%)
**Visual Search**	1.9 (1.0,2.4)	98 (83,100)	1.8 (1.1,3.7)	98 (90,100)	2.4 (1.2,6.0)	95 (13,100)
**Single Road Sign**	1.2 (0.4,1.7)	100 (97,100)	1.5 (0.9,2.8)	97 (94,100)	1.8 (1,5.5.0)	97 (56,100)
**Double Road Sign**	1.7 (0.7,2.4)	92 (86,97)	2.3 (1.2,3.1)	94 (83,100)	2.5 (1.7,6.0)	92 (81,100)

### Correlation with clinical and demographic variables

#### Relationship of task performance with visual function, age and quality of life

**Fig 4:**
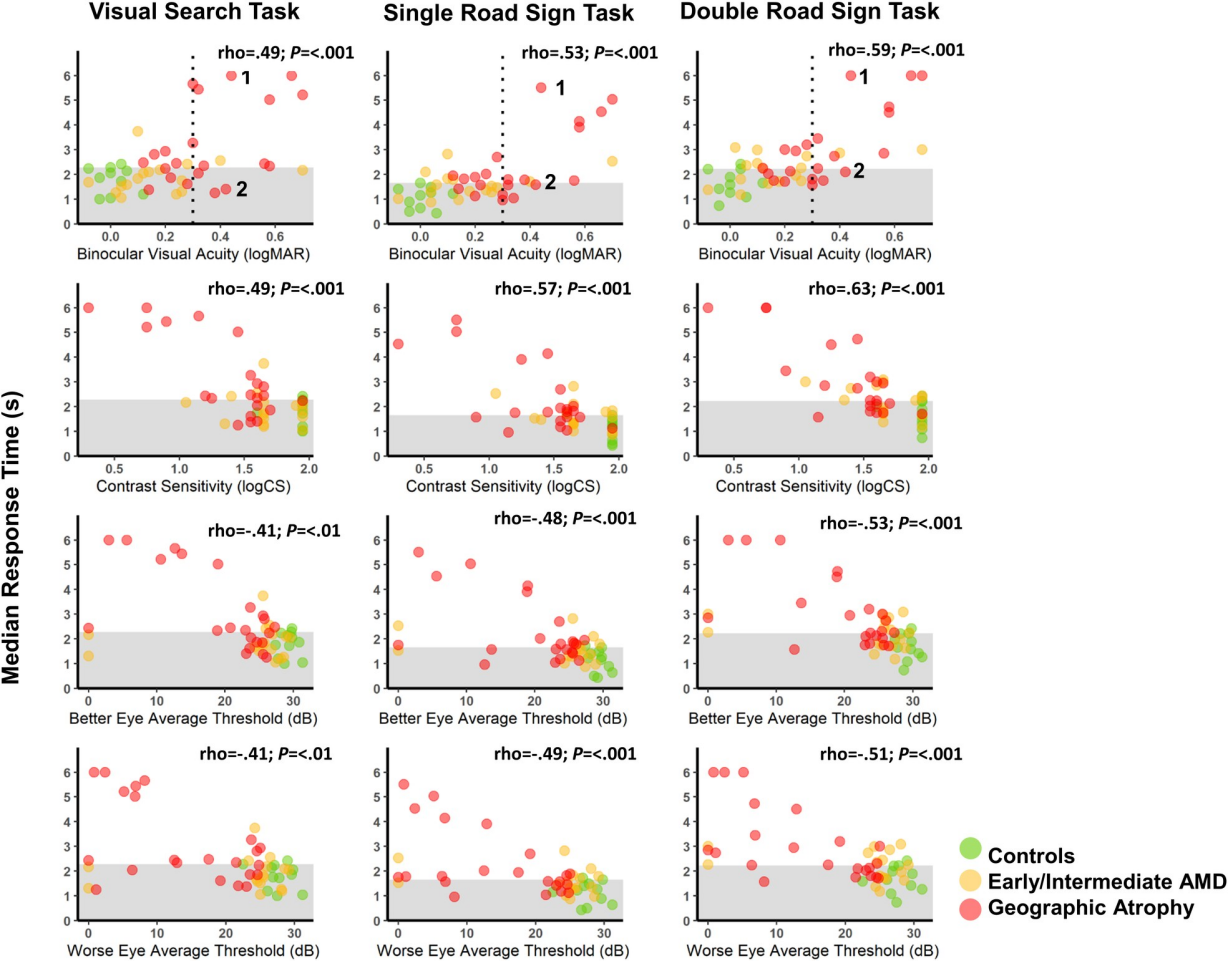
Scatterplots showing relationship between median response time and clinical and demographic variables. Comparing measures of visual function to the median response times (seconds) of the three tasks with respect to the normative limit set at 90% from the control group’s results (shaded area). The average thresholds are calculated from microperimetry data. The dotted, vertical line on the top row of plots shows the minimum visual acuity required to legally drive in the UK (0.3logMAR). Two case studies have been labelled on the visual acuity plots (1–2) to be further discussed.

Varying levels of moderate association was found between RTs and measures of visual function (see Fig 4). The association between TCRs and visual function were generally weaker (S1 Fig). Age was significantly correlated with RT in the road sign tasks (*P* = .01 for both), but not for the visual search task. Age was not significantly correlated with TCR across all tasks. EQ-5D index score was not significantly correlated with RTs or TCRs across all tasks.

### Case studies

Two cases from the GA group who performed quite differently on the tasks have been highlighted (see Fig 4 and S1 Fig). We examined their individual MP results from each eye to determine if these data explained the lack of concordance.

**Fig 5:**
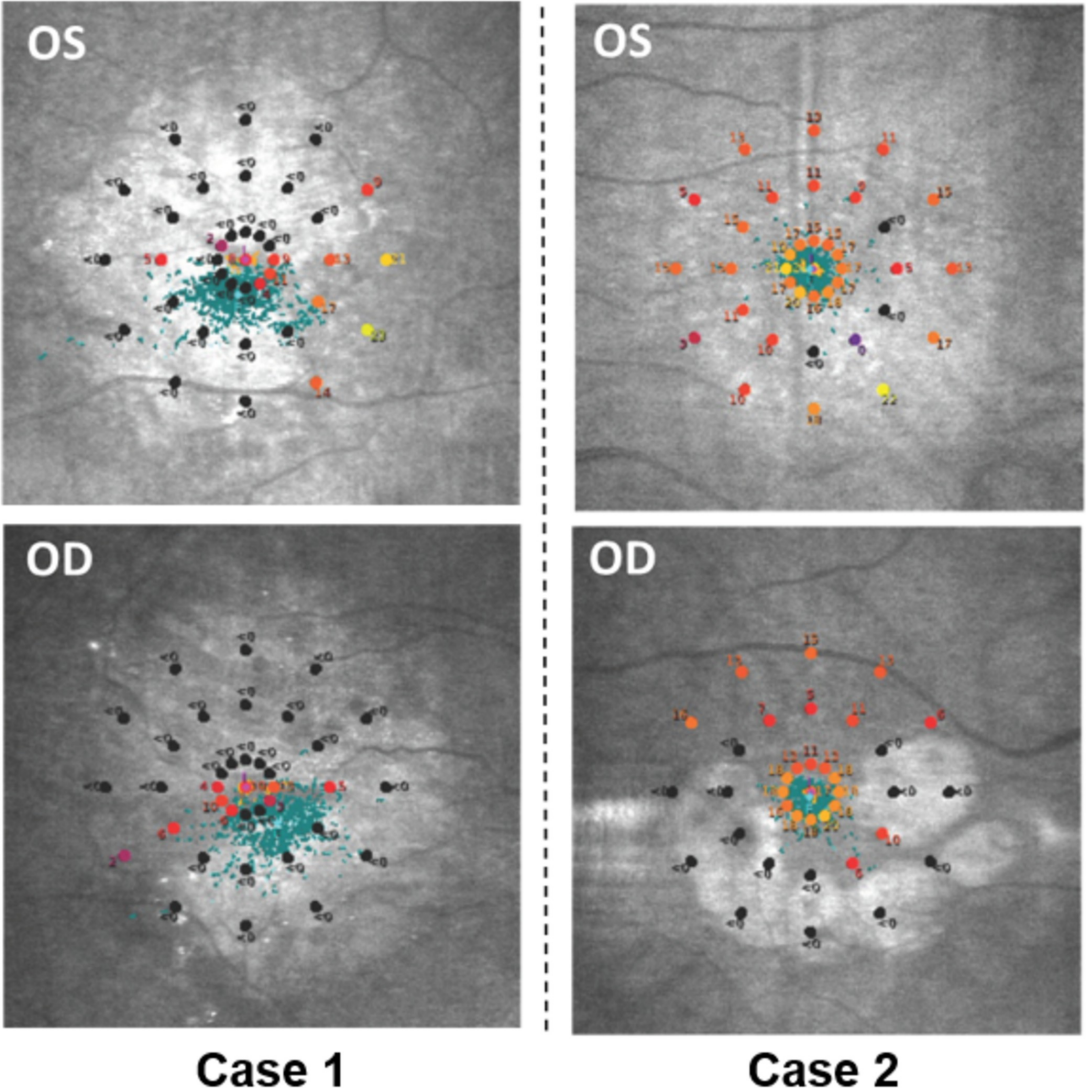
Microperimetry results for cases 1–2. Left eye (OS) and right eye (OD). The blue dots are the fixation points of the eye at time of measurement.

Case 1 (80 year old male) and Case 2 (69 year old male) both have a similar level of BVA (0.42 and 0.44 logMAR, respectively), meaning under current UK law neither are permitted to drive (see Fig 4). Both had a similar level of TCRs in all three tasks. However, Case 1 had poor RT on all tasks and his MP thresholds indicate suspected widespread atrophy and dispersed fixation in both eyes. Case 2 had RTs within the normative range set by the controls. His MP thresholds indicated the least damaged area was the central visual field, with the left eye less damaged than the right. Furthermore, Case 2’s fixation appeared steady compared to Case 1 (see Fig 5).

## Discussion

In this study we investigated measures of visual search performance in people with AMD, using two novel assessments that are more akin to real-world visual experience. The first mimicked search in a busy environment with a task to identify a single everyday object amongst a large number of other objects. The second mimicked an everyday driving scene whereby an approaching road sign must be identified, as if viewed from a moving vehicle. We found statistically significant differences in performance between healthy controls, participants with early/intermediate AMD and participants with GA.

### Slower task performance in severe AMD

Our main finding was that slower performance in these real-world tasks of visual search was associated with more severe AMD. Unsurprisingly, most people with GA performed outside the normative limit on all three tasks. This result is consistent with previous research that people with AMD take longer than visually healthy people to search for real-world targets [5, 9] and in other optotype-based visual tasks [7, 26–28]. Moreover, it agrees with previous research that people with AMD have slower RTs in detection of traffic signals [29, 30]. This may imply a compensatory increase in caution on the road as more time was taken to identify the road signs [14].

We did not find an overall significant difference in the percentage of TCR between people with no AMD and people with AMD in the tasks. It appeared participants with AMD can complete the tasks as well as visually healthy peers, but it takes them longer to do so. Perhaps participants took longer to identify the target images as they may have appeared in their scotoma regions, making it harder for them to locate the images quickly. Although we did not explicitly test for this, MP average thresholds did significantly correlate with TCR for the visual search task and single road sign task. In turn, participants needed longer fixation durations to perform the task effectively [28, 31, 32]. This is supported by studies using simulated scotomas in visual search-based tasks [33] and illustrated by the observations in our two featured case studies. These patients had similar levels of VA but performed differently in the tasks. The MP results of Case 1 illustrated large scotoma that may have compromised the participant’s visual search speed. Case 2 seemed to have a more complete visual field and completed the tasks quickly. Both cases had a similar level of TCR, which suggest Case 2 was not sacrificing task precision for task speed. Interestingly, while Case 2 achieved a marginally higher percentage of TCRs in the static visual search task, this was not the case for the dynamic tasks. This may imply that when the target image moved, it may have been identified by Case 1’s healthier region of the retina and resulted in a correct response. Therefore, MP may give complementary or additional information about the visual function required to carry out real world search activities. Yet all this remains speculation based on these cases and should be the subject of further work; this should be done on a greater sample of participants and ought to include a multivariable analysis.

### Association of task performance with standard measures of visual function

Response time was associated with measures of VA and CS. This is unsurprising and is substantiated in the literature [5, 7, 9, 26, 27, 34–37]. Yet, the equivalent (or marginally higher) levels of association with CS measures is noteworthy. For example, it has been shown that impaired CS has been associated with problems in high-risk driving situations [38] and crash risks [39] compared to visually healthy controls, but has not been exclusively associated with driving safety ratings [15].

Our results provide a useful addition to the literature on driving performance and AMD, highlighting problems that people with AMD may have with the task of identifying and reacting to road signs. Indeed, other research has indicated that people with AMD suffer from insuffiecient observation techniques when driving [15, 30]. Remarkably CS, MP or assessment of visual search is not used to compute minimum vision standards, set by the UK Driver & Vehicle Licensing Agency (DVLA). These guidelines purely rely on VA [40]. However, our data, and the two case studies, suggests that VA alone does not appear to tell a complete story of visual performance for just one aspect of driving, that is timely detecting of road signs.

### Future work and limitations

These tasks were designed to reflect some types of ‘everyday’ visual function. Tasks of this type may feel more meaningful to person’s everyday visual experience compared to imperceptible changes on vision-based charts. Of course, the latter are critical but our tasks could act as potential secondary outcome measures that support measures from clinical charts. In turn we believe they would make an enjoyable and useful addition to clinics, especially if the tests themselves could be performed on portable devices like a tablet computer. We have evaluated the use of the visual search task on a tablet-based platform and the preliminary assessment indicated that it provided a simple, quick and easy‐to‐administer measure of real‐world visual function in healthy volunteers [6]. Our study has indicated that these tasks can be used in people with AMD and older adults. The potential applications include their use in clinic waiting rooms, and as an objective complement to PROMs, especially in a clinical trial setting [6].

Furthermore, a low-contrast or scotopic version of our assessments may provide better discrimination between visually healthy controls and people with AMD. This could be the subject of future work. Moreover, whilst we refer to our assessments having a “real-world” element we recognise they are, for example, dissimilar to everyday visual search tasks with fewer high level elements like depth, overlap, differences in contrast and crowding. Still, we hope to build on the existing literature on image modification in visual search to develop a task that has potential to be used as a secondary outcome measures [41].

We did not use any vision-related PROMs merely using EQ-5D as measure of general health–this is a limitation of our study. While no significant correlations were found with the EQ5D metric and test outcomes, it is possible that a vision-specific PROM may demonstrate different results. Still, discrepancies between task performance and self-reported performance have been previously reported [42] and this is noteworthy. We also did not incorporate measures of participant’s driving ability (experience of adverse driving event, for example) or perception of driving ability—this is another limitation of our study. While these variables may offer interesting additions, our task does not assess driving ability in people per se. We use it as an example of identifying a moving target in a real-world setting. In addition, there has been reported disagreement between self-reported adverse driving events and those officially recorded in elderly populations [43]. Therefore, we chose to compare the test outcomes to frequently used visual function assessments used in clinic e.g. VA, CS and mean sensitivity from MP. A potential option for further work would be to evaluate other MP metrics like pattern standard deviation [44, 45].

Another limitation of this study was that we did not individually compare data from early and intermediate AMD groups. Our sample sizes were too small to do this. Furthermore, 82% of our participants were female. Still, this was an exploratory study and it did yield statistically significant effects in people with more advanced AMD. The tests were conducted in a university research lab and we have now developed versions of the assessment that can be performed on a tablet computer [6] and this is the subject of studies in hospital clinics.

A further limitation of the results may have resulted from the task itself. For example, in the visual search task the target object was not presented simultaneously as the participant searched for it. Although we screened for cognitive loss (participants were required to pass an abridged version of the MMSE [19]) this memory component may have confounded our results. One way to eliminate this potential limitation would be to retain the target image in the centre of the screen throughout the trial. Indeed this is the approach we have taken with the updated version of this this task in other studies [6].

## Conclusions

We have explored the use of computer-based tasks mimicking everyday visual function such as search and identification. We found participants with AMD, especially those with advanced AMD in their better seeing eye, had worse average reaction times on these tasks when compared to visually healthy peers. We found measures of standard visual function were associated with the performance of these tasks. A surrogate task of everyday visual function, like searching for an object or something related driving, is a meaningful reflection of everyday visual experience to a person with AMD. The results from this exploratory study suggest these novel assessments, with further development, could be useful as patient-relevant measures of everyday visual function.

## Supporting information

S1 Video Video demonstration of baseline testing procedure. (MP4)

S2 Video Video demonstration of visual search task. (MP4)

S3 Video Video demonstration of single road sign task. (MP4)

S4 Video Video demonstration of double road sign task. (MP4)

S1 Fig Scatterplots showing relationship between median response time and clinical and demographic variables. Measures of visual function compared to the percentage of total correct responses of the three tasks with respect to the normative limit set at 90% from the control group’s results (shaded area). The average thresholds are calculated from microperimetry data. The dotted, vertical line on the top three plots shows the minimum visual acuity required to legally drive in the UK (0.3logMAR). Two case studies have been labelled on the visual acuity plots (1–2). Significant correlations were found between some measures of visual function and total correct responses, but less so than response time.
(TIF)

S1 Dataset (CSV)
