# Supplementary figures and images for: Novel computer-based assessments of everyday visual function in people with age-related macular degeneration
Source: PLoS One. 2020 Dec 7;15(12):e0243578. doi: 10.1371/journal.pone.0243578 (PMC7721163; doi:10.1371/journal.pone.0243578)

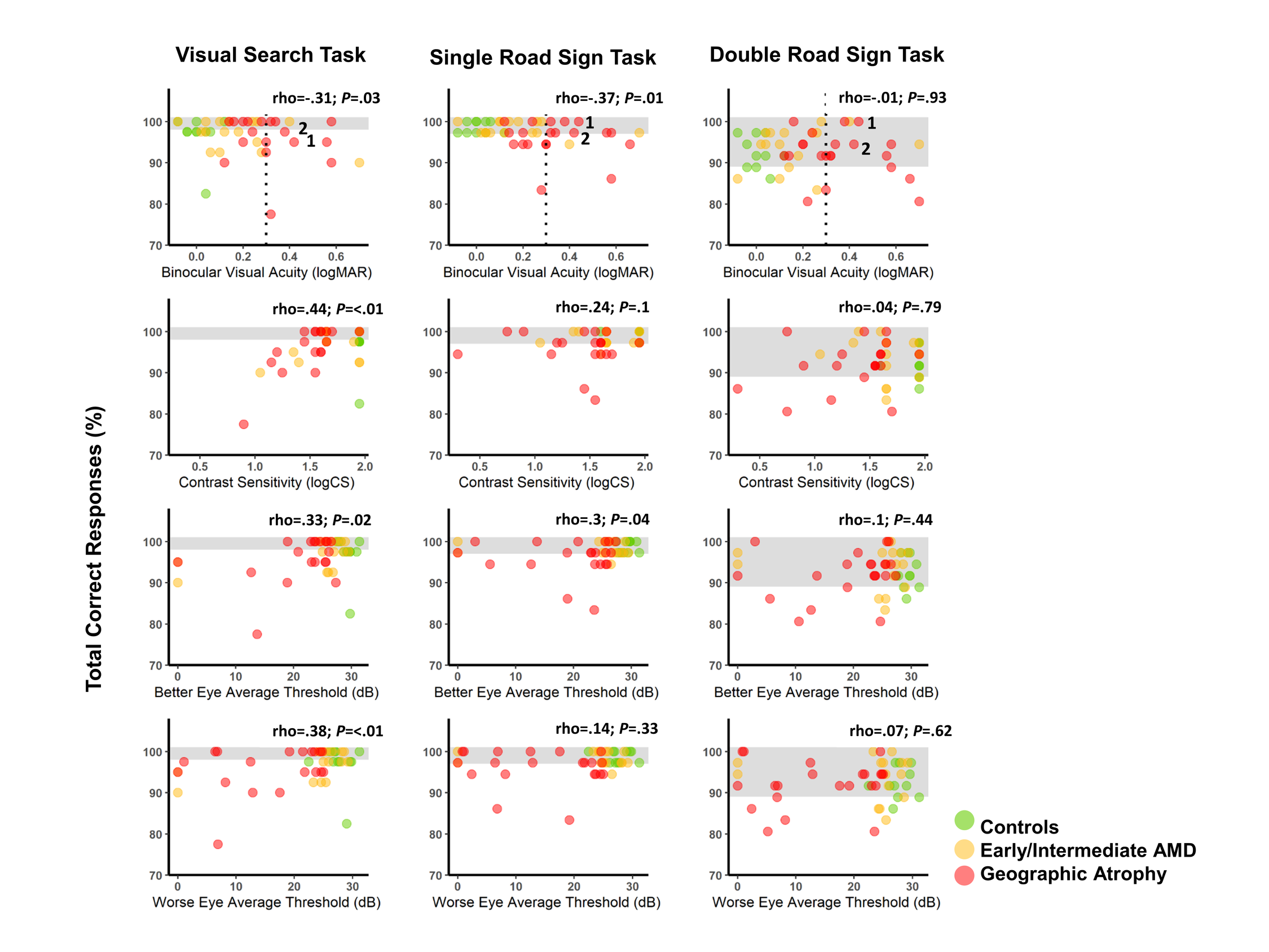

Supplement: S1 Fig — Measures of visual function compared to the percentage of total correct responses of the three tasks with respect to the normative limit set at 90% from the control group’s results (shaded area). The average thresholds are calculated from microperimetry data. The dotted, vertical line on the top three plots shows the minimum visual acuity required to legally drive in the UK (0.3logMAR). Two case studies have been labelled on the visual acuity plots (1–2). Significant correlations were found between some measures of visual function and total correct responses, but less so than response time. (TIF) [file pone.0243578.s005.tif]
